# *In vitro* anthelmintic activity of aqueous and ethanol extracts of *Paraserianthes falcataria* bark waste against *Haemonchus contortus* obtained from a local slaughterhouse in Indonesia

**DOI:** 10.14202/vetworld.2020.1549-1554

**Published:** 2020-08-11

**Authors:** Zein Ahmad Baihaqi, Irkham Widiyono, Wisnu Nurcahyo

**Affiliations:** 1 Student of Doctoral Program Veterinary Science, Faculty of Veterinary Medicine, Universitas Gadjah Mada, Yogyakarta, Indonesia; 2 Department of Internal Medicine, Faculty of Veterinary Medicine, Universitas Gadjah Mada, Yogyakarta, Indonesia; 3 Department of Parasitology, Faculty of Veterinary Medicine, Universitas Gadjah Mada, Yogyakarta, Indonesia

**Keywords:** anthelmintic, bark, *Haemonchus contortus*, prevalence, scanning electron microscopy

## Abstract

**Aim:**

This study was conducted to determine the anthelmintic activity of aqueous and ethanol extracts of *Paraserianthes falcataria* bark against *Haemonchus contortus*.

**Materials and Methods:**

Ethanol extract of bark (E.E.B.) waste and aqueous extract of bark (A.E.B.) waste of *P. falcataria* (at concentrations 0, 0.2, 0.4, 0.8, 1, 2.5, and 5%) and albendazole (2 mg/ml) as the positive control were placed in separate Petri dishes (50 mm). Twenty *H. contortus* worms were placed in Petri dishes and incubated at 37°C for 0.5, 1, 2, 3, 4, 5, 6, and 12 h. Mortality of each worm was ensured by pressing the body of the worm with a pair of tweezers and keeping it in lukewarm water for 5 min before declaring it dead. Mortality is defined as amount of death individuals and time of mortality of each worm was recorded. The parasites were then observed using scanning electron microscopy (SEM) at an accelerating voltage of 15 Kv. Statistical analysis was performed using SPSS 21.0 software, two-way ANOVA followed by Tukey’s test to detect significant differences (p<0.05). The result was expressed as the mean ± standard deviation.

**Results:**

The E.E.B. and A.E.B. of *P. falcataria* contained active compounds, such as tannin, alkaloid, flavonoid, saponin, steroid, and triterpenoid. E.E.B. had a higher content of phenol, while A.E.B. had a higher content of flavonoid. In this study, *P. falcataria* showed a significant effect (p=0.00) on *H. contortus in vitro*. E.E.B. (0.8%) was able to exterminate *H. contortus* completely after 6 h, more effective than A.E.B. (5%) while the positive control requires (2 mg/ml) after 2 h. SEM analysis of the worm treated with E.E.B. and A.E.B. showed damaged cuticle structure.

**Conclusion:**

The aqueous and ethanol extracts of *P. falcataria* bark waste demonstrated anthelmintic activity against *H. contortus*.

## Introduction

*Haemonchus contortus* is one of the most important parasitic nematodes of ruminant livestock worldwide [1]. Cortes-Morales *et al*. [2] stated that *H. contortus* caused large production losses worldwide and worm infestation caused an infection characterized by weight loss, diarrhea, anemia, edema, ill-thrift, acute weakness, and eventually death. *H. contortus* is a blood-sucking parasite found mostly in the small abomasum of ruminant livestock. Niciura *et al*. [3] added that the gastrointestinal nematode significantly affected the sheep livestock and was viewed as the most pathogenic parasite in tropical areas. Baihaqi *et al*. [4] stated that the parasitic worm with the highest prevalence in Wonosobo Regency, Indonesia, was *H. contortus*, followed by *Ostertagia* spp., *Trichostrongylus* spp., *Bunostomum*spp., *Trichuris* spp., and *Moniezia* spp., during the dry and rainy seasons. The tropical environmental conditions in Indonesia constitute an ideal habitat for parasitic species.

Secondary metabolite compounds of plants showed bioactivity toward *H. contortus* which is resistant to the multidrugs given to small ruminants [5]. Zarza-Albarrán *et al*. [6] stated that pressing the nematode showed the presence of phenolic compounds in the plant extracts that were responsible for the anthelmintic effect. Maestrini *et al*. [7] added that secondary metabolite compounds present in plant extracts were responsible for the anthelmintic activity. Carvalho *et al*. [8] added that phenolic compounds, tannins, and flavonoids, such as those in *Stylosanthes guianensis*, were effective against eggs and larvae of gastrointestinal parasites and could be considered as potential alternative naturally anthelmintic treatments. Kommuru *et al*. [9] stated that the worms that interacted with the condensate tannin showed shrunken and tangled cuticle surfaces.

Large wastes from the agricultural industry can pose a severe environmental issue [10]. FAO revealed that approximately 55 million metric tons of waste were produced from processing, packaging, and distribution in the plant production process [11]. Sagar *et al*. [12] stated that plant waste, including seeds, fruit skin, and plant bark containing a number of bioactive compound sources could be used to support the other sectors. Krisnawati *et al*. [13] stated that *Paraserianthes falcataria* is a native plant of the eastern regions of Indonesia (Sulawesi, Papua, and Maluku). Nawir *et al*. [14] stated that the manufacturing industries of *P. falcataria* in Wonosobo Regency rapidly developed due to the high demand of *P. falcataria* in Japan. Roheem *et al*. [15] added that bark waste could be used effectively as alternative medicine because it contains active compounds. King *et al*. [16] stated that *Albizia falcataria* plant contains alkaloids, tannins, flavonoids, saponins, steroids, and triterpenoids.

This research was aimed at determining the anthelmintic potency of *P. falcataria* (Sengon) bark waste with aqueous and ethanol extracts toward *H. contortus* nematodes.

## Materials and Methods

### Ethical approval

This research was approved by the Institutional Ethical Committee, Faculty of Veterinary Medicine, Universitas Gadjah Mada, Yogyakarta, Indonesia. Number: 0013/EC-FKH/Int./2019.

### Study period and location

This study was conducted from October 14, 2019 until November 23, 2019 in Animal Parasitology Laboratorium, Department of Internal Medicine, Faculty of Veterinary Medicine, Universitas Gadjah Mada, Indonesia.

### Plant waste collection and extraction

*P. falcataria* bark waste was collected from the plantation industry in Wonosobo Regency, Central Java, Indonesia. The extraction was performed at room temperature after samples were air-dried and finely powdered using a grinder. The extract used in the treatment was divided into two which were ethanol extract of bark (E.E.B.) and aqueous extract of bark (A.E.B.). One hundred grams of *P. falcataria* bark waste were macerated in water and ethanol for 48 h with intermittent shaking. The extract was then filtered through Whatman filter paper No.1. and concentrated under vacuum on a rotary evaporator [17].

### Determination of plant phytochemistry

To detect the bioactive compounds such as alkaloids, flavonoids, phenols, tannins, steroids, and alkaloids, both the aqueous and ethanol extracts were subjected to qualitative phytochemical screening using standard procedures [18]. The total phenolic content in the plant waste extracts was measured using Folin–Ciocalteu method and the results were expressed as mg gallic acid equivalents [19], while the total flavonoid content of plant waste extracts was determined using colorimetric method and the results were expressed as mg rutin (RE), as previously reported [20].

### *In vitro* adult worm mortality test

The adult female worms of *H. contortus* were collected from the slaughterhouse for sheep in Godean, Yogyakarta. The *in vitro* anthelmintic study was modified [21]. The aqueous and ethanol extracts of *P. falcataria* bark waste (0, 0.2, 0.4, 0.8, 1, 2.5, and 5%) were placed in a Petri dish (50 mm); 20 adult worms of *H. contortus* were incubated at 37°C for 0.5, 1, 2, 3, 4, 5, 6, and 12 h, using 2 mg/ml albendazole (Kimia Farma, Indonesia) as the positive control. The procedure was performed daily for 3 days. The death of the worm was secured by pressing the body of the worm using a pair of tweezers and keeping it in lukewarm water for some time before declaring it dead. Mortality is defined as amount of death individuals and time of mortality of each worm was recorded.

### Scanning electron microscopy (SEM)

The worms obtained from the *in vitro* assay studies were fixed with 2% glutaraldehyde solution with sodium cacodylate as buffer (0.1 M) for 4 h at 4°C. After two washes in the same buffer (0.2 M), the worms were dehydrated in a graded ethanol series, dried by critical point drying with EMSCOPE CPD 750, and coated with gold-palladium for 5 min at 100 Å/min. The parasites were then observed with S450 scanning electron microscope (Hitachi) at an accelerating voltage of 15 Kv [22].

### Statistical analysis

The results of *in vitro* mortality of *H. contortus* were recorded and analyzed by SPSS 21.0. We performed a two-way ANOVA followed by Tukey’s test to detect significant differences (p<0.05). The result was expressed as the mean ± standard deviation.

## Results

The presence of the two solvents showed a positive effect qualitatively on secondary metabolite compounds such as tannins, flavonoids, alkaloids, saponins, and steroids (Table-1).

**Table-1:** Qualitative phytochemical analysis.

Secondary metabolite	Bark of *Paraserianthes falcataria*	

	Aqueous	70% ethanol
Tannin	+	+
Flavonoid	+	+
Alkaloid	+	+
Saponin	+	+
Steroid	**+**	**+**

Table-2 shows the total phenol and flavonoid contents in *P. falcataria* bark wastes with aqueous and 70% ethanol extracts. The highest total phenolic content was observed in E.E.B. *P. falcataria* (10 mg GAE/g dw) while the highest total flavonoid content was observed in A.E.B. *P. falcataria* (3.3 mg RE/g dw).

**Table-2:** Total phenol and flavonoid contents in the studied plant in mg/g dry weight.

Plant extract	Total phenolic (mg GAE/g dw)	Flavonoids content (mg RE/g dw)
E.E.B. *Paraserianthes falcataria*	10	1.6
A.E.B. *Paraserianthes falcataria*	7.5	3.3

E.E.B: Ethanol extract of bark, A.E.B: Aqueous extract of bark

Table-3 shows that E.E.B. *P. falcatari*a significantly (p=0.00) caused the death of *H. contortu*s *in vitro*, starting at treatments 0.2% after 3 h, while the A.E.B. *P. falcataria* was observed to start inhibiting at a concentration of 0.2% at 2 h into the experiment (Table-4). Positive control (albendazole) was observed to start worm death in the 1^st^ half an hour by 61.67±15.28 and 100% worm death by the 2^nd^ h. E.E.B. *P. falcataria* was controlled to have 100% lethal ability toward *H. contortu*s at a lower concentration (0.8%) than A.E.B. (5%).

**Table-3:** *In vitro Haemonchus contortus* mortality test (E.E.B. *P. falcataria).*

Treatment (%)	Time of death (h) – E.E.B. *P. falcataria*								SEM	p-value

	0.5	1	2	3	4	5	6	12		
0	0.00±0.00^C^	0.00±0.00^C^	0.00±0.00^E^	0.00±0.00^E^	0.00±0.00^D^	0.00±0.00^D^	0.00±0.00^C^	0.00±0.00^C^	0.00	-
0.2	0.00±0.00^Ce^	0.00±0.00^Ce^	0.00±0.00^Ee^	21.67±2.89^Dd^	33.33±5.77^Cc^	41.67±2.89^Cc^	60.00±5.00^Bb^	71.67±2.89^Ba^	5.51	0.000
0.4	0.00±0.00^Cd^	8.33±5.77^Ccd^	13.33±7.64^DEc^	18.33±2.89^Dc^	35.00±5.00^Cb^	45.00±5.00^Cb^	63.33±2.89^Ba^	68.33±2.89^Ba^	5.06	0.000
0.8	0.00±0.00^Cf^	8.33±2.89^Ce^	21.67±2.89^De^	43.33±7.64^Cd^	63.33±10.41^Bc^	81.67±7.64^Bb^	100±0.00^Aa^	100.00±0.00^Aa^	7.87	0.000
1	3.33±5.77^Ce^	11.67±2.89^Ce^	43.33±12.58^Cd^	70.00±0.00^Bc^	80.00±8.66^Bbc^	91.67±2.89^Aab^	100.00±0.00^Aa^	100.00±0.0^Aa^	7.62	0.000
2.5	8.33±2.89^BCd^	23.33±7.64^BCd^	51.67±7.64^Cc^	73.33±7.64^Bb^	81.67±12.58^ABab^	100.00±0.00^Aa^	100.00±0.00^Aa^	100.00±0.00^Aa^	7.12	0.000
5	16.67±7.64^Bd^	43.33±15.28^ABc^	75.00±13.23^Bb^	100.00±0.00^Aa^	100.00±0.00^Aa^	100.00±0.00^Aa^	100.00±0.00^Aa^	100.00±0.00^Aa^	6.46	0.000
Albendazole 2 mg/ml	31.67±7.64^Ac^	61.67±15.28^Ab^	100.00±0.00^Aa^	100.00±0.00^Aa^	100.00±0.00^Aa^	100.00±0.00^Aa^	100.00±0.00^Aa^	100.00±0.00^Aa^	5.17	0.000
SEM	2.35	4.55	7.18	7.48	7.10	7.26	7.02	6.84	-	-
p-value	0.000	0.000	0.000	0.000	0.000	0.000	0.000	0.000	-	-

**Table-4:** *In vitro Haemonchus contortus* mortality test (A.E.B.* P. falcataria).*

Treatment (%)	Time of death (h) – A.E.B. *P. falcataria*								SEM	p-value

	0.5	1	2	3	4	5	6	12		
0	0.00±0.00^C^	0.00±0.00^C^	0.00±0.00^F^	0.00±0.00^E^	0.00±0.00^F^	0.00±0.00^F^	0.00±0.00^E^	0.00±0.00^E^	0.00	-
0.2	0.00±0.00^Ce^	0.00±0.00^Ce^	10.00±5.00^DEFd^	13.33±2.89^Dd^	16.67±2.89^EFcd^	23.33±2.89^Ebc^	30.00±5.00^Db^	41.67±2.89^Da^	2.87	0.000
0.4	0.00±0.00^Cd^	0.00±0.00^Cd^	8.33±2.89^EFd^	20.00±5.00^Dc^	25.00±5.00^Ebc^	35.00±5.00^DEb^	55.00±5.00^Ca^	58.33±2.89^Ca^	4.51	0.000
0.8	0.00±0.00^Cg^	5.00±8.66^BCfg^	11.67±2.89^DEef^	20.00±0.00^De^	31.67±2.89^DEd^	45.00±5.00^Dc^	63.00±2.89^Cb^	78.33±2.89^Ba^	5.56	0.000
1	6.67±2.89^BCf^	11.67±7.64^BCf^	20.00±5.00^CDde^	36.67±2.89^Cd^	56.67±10.41^BCc^	73.33±7.64^BCbc^	81.67±7.64^Bab^	95.00±5.00^Aa^	6.69	0.000
2.5	11.67±2.89^Be^	16.67±2.89^BCe^	30.00±5.00^BCd^	38.33±2.89^Cd^	50.00±5.00^CDc^	60.00±5.00^Cc^	76.67±2.89^Bb^	96.67±2.89^Aa^	5.77	0.000
5	16.67±5.78^Bd^	23.33±7.64^Bd^	35.00±5.00^Bcd^	51.67±7.64^Bc^	75.00±13.23^Bb^	85.00±8.66^Bab^	100.00±0.00^Aa^	100.00±0.00^Aa^	6.69	0.000
Albendazole 2 mg/ml	31.67±7.64^Ac^	61.67±15.28^Ab^	100.00±0.00^Aa^	100.00±0.00^Aa^	100.00±0.00^Aa^	100.00±0.00^Aa^	100.00±0.00^Aa^	100.00±0.00^Aa^	5.17	0.000
SEM	2.31	4.25	6.23	6.05	6.48	6.56	6.79	7.02	-	-
p-value	0.000	0.000	0.000	0.000	0.000	0.000	0.000	0.000	-	-

## Discussion

Qualitatively, the phytochemical analysis on the aqueous and 70% ethanol extracts of *P. falcataria* bark waste was found positive for active compounds in the form of tannins, flavonoids, alkaloids, saponins, and steroids. The results of a study conducted by Raipuria *et al*.[23] showed that the aqueous and ethanol extracts used in the analysis of active compounds in plants were qualitatively equal and *in vivo* assays could be used for reference. Amadioha and Chidi stated that aqueous and ethanol extracts qualitatively showed no differences when analyzed for active compounds in plants. Active phytochemicals are naturally produced by plants to help them suppress diseases or pathogenic organisms [24]. Cheruiyot *et al*. [25] added that phytochemical screening of plants was used to determine potentially active compounds in plants.

Phenol compounds are natural active secondary metabolites of plants that can be used as a defense to protect the plant from parasitic infections [26]. Montoro *et al*. [27] stated that flavonoids are naturally found in plants and are believed to have several effects on plant health. The studies on flavonoid derivatives proved that flavonoids have antibacterial, anthelmintic, antiviral, anti-inflammatory, anti-allergic, and anticancer activities. Plant active compounds were proved to suppress *H. contortus* [28-31]. Badar *et al*. [32] stated that the variation in anthelmintic activity *in vitro* could be affected by the type of solvent extracts of plants and the difference in the content of active compounds in bark of plants.

The first 100% inhibition and suppression of *H. contortus* was monitored faster for ethanol extract than aqueous extract of *P. falcataria*. These results were the same as the findings of Kumari *et al*. [33] stated that anthelmintic observations using solvent extracts of *Azadirachta indica* were more easily monitored because 100% suppression required only 2 g/l of ethanol extract and 3.5 g/l of aqueous extract. At concentration 1 g/l, ethanol extract showed 50% suppression while aqueous extract still showed 0% suppression [33]. Cala *et al*. [34] stated that *in vitro* observations of the inhibition of anthelmintic effects toward *H. contortus* by the utilization of secondary metabolite compounds in plants were influenced by the type of solvent used, the dosage, and the type of plant used.

**Figure-1:**
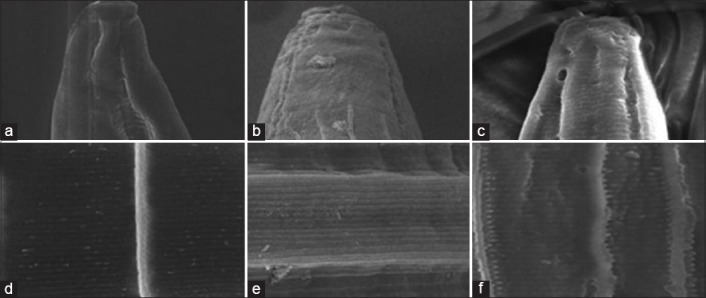
Scanning electron microscopy image of the anterior end and the cuticle of the adult female *Haemonchus contortus*. (a) and (d) The normal fresh worm; (b) and (e) the worm after incubation with E.E.B.; and (c) and (f) the worm after incubation with A.E.B.

Figure-1 shows that the observed change in SEM images in the *in vitro* experiment revealed the interaction between E.E.B and A.E.B. Control worms (a and b) were observed to be smooth, free of aggregates on the cuticle, and having an undamaged reticular longitudinal back, whereas worms that received treatments (b, c, e, and f) were observed to be damaged at the buccal area and show aggregate buildup in the annular cuticles. Findings from research by Acevado-Ramírez *et al*.[21] showed that the effects of anthelmintic activity of *Castanea sativa* on *H. contortus* were destruction around the mouth, anus, vulva, and bursa copulatrix, and loss of cuticle structure integrity in the mid-section of the body coupled with expulsion of digestive tract components. Tresia *et al*. [35] stated that anthelmintic activity was synergistically carried out by active compounds of plant by damaging the cuticle and changing the shape of the pore and permeability of the worm cuticle. Sambodo *et al*. [36] added that cuticle changes due to interactions with aqueous extract of *Biophytum petersianum*, where the part has an important role in the motility and absorption of nutrients.

SEM results revealed severe damage to the cuticle of *H. contortus* after contact with *Leucosidea sericea* leaf extract and agrimol G component [37]. Yoshihara *et al*. [38] added that the structural changes in the cuticle caused nutritional disorders in the nematodes that could eventually lead to malnutrition. Olivas-Aguirre *et al*. [39] added that the mechanism of damage to body parts of the nematodes on contact with active plant compounds was by the formation of complex collagen-tannins due to the high content of proline in collagen or nematode cuticle damage by secondary metabolite compounds in plants.

## Conclusion

This research showed that the aqueous and ethanol extracts of *P. falcataria* bark waste contained tannins, flavonoids, alkaloids, saponins, and steroids and were, hence, able to deactivate *H. contortus*. This was proved from the results of the mortality test and the structural changes and damage to the cuticle and the longitudinal reticular back of the worms.

## Authors’ Contributions

ZAB, IW, and WN designed the study. ZAB conducted the field survey. ZAB, IW, and WN collected samples and examined in the laboratory. All authors drafted and revised the manuscript. All authors have read and approved the final manuscript.

## Competing Interests

The authors declare that they have no competing interests.

## Publisher’s Note

Veterinary World remains neutral with regard to jurisdictional claims in published institutional affiliation.
